# The association between ethnicity, stigma, beliefs about medicines and adherence in people living with HIV in a rural area in Indonesia

**DOI:** 10.1186/s12889-019-6392-2

**Published:** 2019-01-11

**Authors:** E. I. Sianturi, D. A. Perwitasari, Md. A. Islam, K. Taxis

**Affiliations:** 1 PharmacoTherapy, Epidemiology & Economics (PTEE) Department of Pharmacy, University of Groningen, Antonius Deusinglaan 1, 9713 AV Groningen, The Netherlands; 2 Faculty of Mathematics and Natural Sciences, University of Cenderawasih, Papua, Indonesia; 3 Faculty of Pharmacy, University of Ahmad Dahlan, Yogyakarta, Indonesia; 4 Department of Statistics, Shahjalal University of Science and Technology, Sylhet, Bangladesh

**Keywords:** HIV, Adherence, Stigma, Beliefs, Indigenous, Indonesia

## Abstract

**Background:**

Indonesia is one of Asia’s countries with the fastest growing rate of human immunodeficiency virus (HIV) infections according to the World Health Organization (WHO). The prevalence of HIV infections in the province of Papua is 2.4% which is 24 times higher than the national rate in Indonesia. This study aimed to investigate the association between stigma, beliefs about medicines, sociodemographic characteristics including ethnicity and adherence in People living with HIV (PLHIV) in Papua, Indonesia.

**Methods:**

We conducted a cross-sectional study using questionnaires. We included participants from two hospital-outpatient clinics who were on antiretroviral treatment (ART) for more than 6 months, were at least 18 years old, and signed informed consent. Participants completed the Medication Adherence Rating Scale (MARS), Beliefs about Medicines Questionnaire (BMQ), an HIV stigma scale and questions on demographic information. Data on antiretroviral medications were collected from medical records. The outcome was self-reported adherence as measured by the MARS using an 80% cut-off score. Multivariate logistic regression was used to analyse the data.

**Results:**

Overall, 331 out of 363 eligible participants were included with a mean age of 33.3 (± 9.4) years, 61.6% were female, 67.1% were Papuan. A total of 65.9% of participants were adherent. Being Papuan decreased the likelihood of adherence (odds ratio (OR) = 0.53; 95% confidence interval (CI) = 0.32–0.89). Feeling more distant, a stigma type, also decreased the likelihood of adherence (OR = 0.93; 95% CI = 0.88–0.99).

**Conclusion:**

The ethnicity of being Papuan and taking a distance to others were associated with non–adherence. Targeted interventions should be developed to improve adherence in this group.

**Electronic supplementary material:**

The online version of this article (10.1186/s12889-019-6392-2) contains supplementary material, which is available to authorized users.

## Background

More than 36 million people who are infected with the human immunodeficiency virus (HIV) worldwide require antiretroviral therapy (ART) to reduce mortality and the number of new HIV infections [1]. ART is a lifetime medication and adherence to treatment is necessary to be effective [2]. Based on a definition by the World Health Organization (WHO), we understand adherence to be the extent to which a person’s behavior in taking medication corresponds with agreed recommendations from a health care provider [3]. Stigma, family responsibilities, and problems with schedule and routine have been recognized as barriers to adherence [4].

Stigma is understood as a society’s attitude in disgracing a person or group [5]. Feeling stigmatized is very common in people living with HIV (PLHIV). PLHIV can notice the stigma resulting in marginalizing behavior towards the PLHIV [6]. It has been shown that stigmatized PLHIV were less able to manage their disease [7]. Previous studies have also shown that stigma was a significant predictor for a lower level of ART adherence in developing and developed countries [8].

Beliefs about Medicines is a concept of cognitive representation of medicines which has been introduced by Horne et al. [9]. They developed a questionnaire to quantify people’s beliefs of the necessity of taking medications versus concerns about adverse effects. In particular, having concerns about adverse effects and not being convinced of the necessity of the medicines have been found to be associated with non-adherence to ART [10]. Providing information and communicating about the complexity of ART and possible adverse effects may improve adherence [11].

Papua is the easternmost island of Indonesia. In the province of Papua, the majority of the population are indigenous Papuans. Ethnic non-Papuans are migrants from other Indonesian islands, mostly to seek employment. Indonesia is one of Asia’s countries with the fastest growing rate of HIV infections according to the Indonesian Ministry of Health and WHO. The prevalence of HIV infections in the province of Papua is 2.4% which is 24 times higher than the national rate in Indonesia. Of the 25.000 people living with acquired immune deficiency syndrome (AIDS) in Papua [12], less than 6000 PLHIV are on ART [13]. The main mode of transmission is through unprotected sex between heterosexuals [12]. In contrast to other areas in Indonesia, most of the inhabitants of Papua are Christians.

In 2014, the Indonesian government set the strategic target to have 80% of PLHIV covered by ART over 5 years [14]. This may effectively decrease the epidemic. Maintaining adherence to ART has been regarded as a major component to achieve this goal. A number of studies have investigated the level of ART adherence in Indonesia, but so far, these merely covered urban cities in Indonesia with a different sociodemographic and epidemiological context to Papua [15, 16]. Only one Indonesian study explored the effect of stigma, again in an urban population not using a validated scale to assess stigma [16]. As far as we are aware, beliefs about medicines have not been studied systematically in Indonesia. Understanding which factors are associated with low medication adherence is the base to develop interventions to improve treatment of HIV in rural areas like Papua [17].

This study aimed to investigate the association between stigma, beliefs about medicines, sociodemographic characteristics including ethnicity and adherence in people living with HIV in Papua, Indonesia.

## Methods

### Study design, setting and participants

This was a cross-sectional study using questionnaires to assess stigma, beliefs about medicines and adherence to medication. The participants were recruited from the outpatient clinics of one public hospital and one private hospital in Jayapura. These are two out of three hospitals providing HIV care in Jayapura. We have selected those two hospitals as they were the first ones to provide HIV care by teams of specialists for 10 years. ART is dispensed free of charge to patients.

Participants were included if they were at least 18 years old, were on ART for more than six months, and signed informed consent. Patients were excluded if more than 50% of data was missing. Participants were recruited and informed about the study while they were collecting their ART in the hospital. The recruiters, one pharmacist and three nurses who were health care professionals from the two HIV study hospitals, explained the aim of the study. They also emphasized that all information was kept confidential and that the decision whether to participate or not did not affect their treatment in any way. After the participants signed the informed consent, an appointment to complete the questionnaires was made for the following month as part of their next visit to the hospital.

The sample size was calculated based on the use of a two tailed test with a level of significance of 0.05. It was assumed that approximately 77% of the population were adherent [16]. Therefore, at least 284 participants were required to conduct the study.

### Instruments

We have chosen to assess adherence [18], beliefs about medicines [9] and stigma [19] with widely used instruments [10, 20, 21].

#### Adherence

We used the self–reported Medication Adherence Rating Scale (MARS) [18]. This consists of 10 questions. This questionnaire assessed the patients’ medication taking behavior in the past week. An example of a statement is: “I take my medication only when I am sick”. The response is binary, yes/no. A total MARS score is calculated as the sum of all items with a maximum score of 10 [18]. A higher total score indicated higher levels of adherence. We dichotomized the response into adherence and non-adherence. Sensitivity analyses were performed with MARS scores of 7, 8 and 9 as the cut-off (Additional file 1: Appendix). The cut-off score of 8 was the most sensitive and was therefore used for the analysis.

#### HIV stigma

The HIV Stigma-Sowell scale consists of 13 questions. These 13 items represent 3 types of stigma, namely distancing, blaming and discrimination [22]. A question representing distancing is: “I thought other people were uncomfortable being with me”. A question representing blaming is: “I felt I would not get good health care if people knew about my illness”. A question representing discrimination is: “I felt compelled to change my residence because of my illness”. Answers are scored on a 4 point Likert scale (as: 1 = not at all, 2 = rarely, 3 = sometimes and 4 = often.) The distancing and blaming scales were assessed with 4 questions each. The discrimination scale was assessed with 5 questions. The total score ranges from 13 to 52 with a higher total score indicating a higher level of stigma.

#### Beliefs about medicines questionnaire (BMQ)

The BMQ assesses patient’s concepts about medication use in general and patient’s beliefs about the medication they use [9]. The BMQ-General part of the questionnaire consists of two scales asking about their views on overuse and harm related to medication (four questions each). An example of a statement on overuse is: “doctors use too many medicines”. An example of a statement on harm is: “most medicines are addictive.” The BMQ-Specific part consists of two scales about the necessity and concerns of patients regarding their medicine (5 questions each). An example of a statement on necessity is: “my antiretroviral medication protects me from becoming worse”. An example of a statement on concern is: “I am sometimes worried about becoming too dependent on antiretroviral medication”. All 18 statements were scored on a 5-point Likert scale as 1 = strongly disagree, 2 = disagree, 3 = uncertain, 4 = agree, 5 = strongly agree. The total from every scale of the BMQ was calculated. Higher total scores on necessity indicated patients being positive and seeing the advantages taking their medication. Higher total scores on concern, overuse, and harm indicated concerns about the treatment.

### Translation

Since all instruments were developed and validated in English, we translated the instruments forwards into Bahasa Indonesia and backwards into English. The forward translation was done by two Indonesian certified translators. Both versions were assessed by DPA as proofreader and reviewer with a lot of experience in translating questionnaires. A final, reconciled Bahasa version was agreed on. This version was back-translated by an English native speaker who had no information about the original versions of the questionnaires. The backward translation was modified several times because the target language does not recognize verb tenses [23]. Therefore, the final version included words related to time. The final questionnaires were tested in 67 PLHIV who did not participate in the main study. The Cronbach’s alpha coefficient to test the internal consistency of the questionnaires was: 0.94 for the MARS scale, 0.82 for the HIV stigma scale and 0.6 for the BMQ scale, indicating good to moderate consistency. Participants indicated they understood the questionnaires.

### Data collection

At the appointment, participants were handed out the three paper-based questionnaires and were also given a questionnaire to collect sociodemographic information (age, gender, marital status, whether or not they had children, education, employment, sexual orientation, religion, ethnicity, receiving support in medicine-taking from social network, and ART regimen). They completed the questionnaires in the waiting area of the hospital. The recruiters were available for questions. Information about the age and types of ART were collected from PLHIV’s medical records. Data were collected between September and November 2016.

### Descriptive statistics

The sociodemographic variables were analyzed using descriptive statistics. The categorical variables were presented as frequencies and percentages. The continuous variables were summarized either as mean (standard deviation) or median (interquartile range) depending on the nature of the variables. The outcome variable was binary coded as non-adherent and adherent. Chi-square test for categorical and independent sample t-test or Mann-Whitney U test were used to determine the association between the independent variables and adherence. We included the following independent variables in the analyses: age, gender, marital status, having children, education, employment, sexual orientation, religion, ethnicity, social support in medicine-taking, ART regimen, distancing, blaming and discrimination of the stigma scale and necessity, concern, overuse and harm from the BMQ questionnaire.

### Statistical modelling

We included all independent variables with a *p* value of < 0.20 in the descriptive analysis in a univariate logistic regression to examine the effect of the independent variables on adherence. Then, independent variables with a *p* value of < 0.20 from the univariate analysis were included in the multivariate logistic regression analysis [24]. In the multivariate logistic regression analysis, we used a backward elimination procedure to select the final model with all independent variables being significant with a *p* value of ≤0.05. Finally, odds ratios of independent variables and their 95% confidence interval were presented. All analyses were done using two-tailed tests at a significance level of 0.05. The statistical analyses were performed using the Statistical Program for Social Sciences (SPSS) version 24.0 for Windows.

## Results

### Study population

We identified 1305 PLHIV starting ART since 2006, successfully contacted and screened 923 PLHIV of whom 388 were eligible. Finally, 363 participants agreed to sign the informed consent and completed the questionnaire (response rate 93%). Thirty-two participants had to be excluded. Two participants were excluded because on inspection of their medical records, they were below 18 years of age and 30 participants were excluded because of missing sociodemographic data. Finally, 331 participants were eligible for data analysis.

#### Sociodemographic characteristics of participants

The mean age of participants was 33.3 years (SD = 9.4), 61.6% were female and 67.1% had a Papuan ethnicity. Almost all participants were educated (51.7% had a vocational and 21.1% had a high school education). More than half of the participants (58.0%) reported having paid employment. The majority (96.4%) were heterosexual, 0.9% were bisexual, and 2.7% were gay. The majority reported Protestantism as their religion (74.6%). A fixed dose combination (FDC) was used as a single tablet ART by 43.5% of all participants*.* The majority of participants (65.9%) received support in medicine-taking from their social network (Table 1).

**Table 1:** Socio-demographic characteristics of participants for adherent vs non-adherent participants

Characteristics	Total (*n* = 331)	Non-adherent (*n* = 113; 34.1%)	Adherent (*n* = 218; 65.9%)	*p*-value
Age (mean, SD)	33.3 (9.4)	31.3 (9.4)	34.3 (9.2)	
18–27 years		43 (38.1)	53 (24.3)	0.019
28–37 years		44 (38.9)	91 (41.7)	
Above 38 years		26 (23.0)	74 (33.9)	
Gender				0.202
Female	204 (61.6)	75 (66.4)	129 (59.2)	
Male	127 (38.4)	38 (33.6)	89 (40.8)	
Marital Status				0.331
Not-married	104 (31.4)	31 (27.4)	73 (33.5)	
Married	200 (60.4)	70 (61.9)	130 (59.6)	
Divorced/widowed	27 (8.2)	12 (10.6)	15 (6.9)	
Having Children				0.259
No	101 (30.5)	30 (26.5)	71 (32.0)	
Yes	230 (69.5)	83 (73.5)	147 (67.4)	
Education				0.309
Elementary school	7 (2.1)	1 (0.9)	6 (2.8)	
Junior high school	28 (8.5)	7 (6.2)	21 (9.6)	
High school	70 (21.1)	30 (26.5)	40 (18.3)	
Vocational	171 (51.7)	57 (50.4)	114 (52.3)	
University	8 (2.4)	4 (3.5)	4 (1.8)	
Master	47 (14.2)	14 (12.4)	33 (15.1)	
Employment				0.076
Unpaid	139 (42.0)	55 (48.7)	84 (38.5)	
Paid	192 (58.0)	58 (51.3)	134 (61.5)	
Sexual Orientation				0.335
Homosexual	9 (2.7)	1 (0.9)	8 (3.7)	
Bisexual	3 (0.9)	1 (0.9)	2 (0.9)	
Heterosexual	319 (96.4)	111 (98.2)	208 (95.4)	
Religion				0.464
Protestant	247 (74.6)	88 (77.9)	159 (72.9)	
Catholic	22 (6.6)	8 (7.1)	14 (6.4)	
Moslim	62 (18.7)	17 (15.0)	45 (20.6)	
Ethnicity				0.023
Non-Papuan	109 (32.9)	28 (24.8)	81 (37.2)	
Papuan	222 (67.1)	85 (75.2)	137 (62.8)	
Support in medicine-taking from social network				0.280
No support	113 (34.1)	43(38.1)	70 (32.1)	
Support	218 (65.9)	70 (61.9)	148 (67.9)	
Regimen				0.248
Duv-EFV	9 (1.2)	1 (0.9)	8 (3.7)	
Duv-NEV	29 (2.7)	7 (6.2)	22 (10.1)	
FDC	144 (43.5)	48 (42.5)	96 (44.0)	
TDF-3TC-Aluvia	2 (0.6)	1 (0.9)	1 (0.5)	
TDF-3TC-EFV	59 (17.8)	18 (15.9)	41 (18.8)	
TDF-3TC-NEV	10 (3.0)	2 (1.8)	8 (3.7)	
TDF-FTC-Aluvia	3 (0.9)	1 (0.9)	2 (0.9)	
TDF-FTC-EFV	70 (21.1)	33 (29.2)	37 (17.0)	
TDF-FTC-NEV	1 (0.3)	0 (0.0)	1 (0.5)	

*SD Standard Deviation, *Duv* Duviral, *EFV* Efavirenz, *NEV* Nevirapine, *FDC* Fixed Dose Combination (TDF + 3TC + Efavirenz), *TDF* Tenofovir, *3TC* Lamivudine, *FTC* Emtricitabine

*p value according to Chi square test

Overall, 65.9% of participants reported to be adherent. There were no significant differences in gender, marital status, having children, education, employment, sexual orientation, religion, and type of regimen between non-adherent and adherent participants. Significant differences were observed in age and ethnicity between adherent and non-adherent participants (Table 1).

Table 2 shows that the most common reasons for being non-adherent were forgetting to take ART (72.1%), carelessly taking ART (51.4%) and attitudes towards medication (55.9%). The difference between domain of BMQ and Stigma scale compared to adherence were shown in Table 3.

**Table 2:** Medication Adherence Rating Scale (MARS) questionnaire (*N* = 113 non-adherent participants)

No	The MARS question	Frequency of responses indicating non-adherence (%)	
		N	(%)
1	Do you ever forget to take your medication?	80	72.1
2	Are you careless at time about taking your medication?	57	51.4
3	When you feel better, do you sometimes stop taking your medication?	40	36.0
4	Sometimes if you feel worse when you take the medication, do you stop taking it?	33	29.7
5	I take medication only when I am sick	39	35.1
6	It is unnatural for my mind and body to be controlled by medication	62	55.9
7	My thoughts are clear on medication	31	27.9
8	By staying on medication, I can prevent getting sick	33	29.7
9	I feel weird, like a “zombie” on medication	45	40.5
10	Medication makes me feel tired and sluggish	43	38.7

**Table 3:** Medians and IQR for the scales used to assess participant’s stigma, and Beliefs about Medicines (BMQ) comparing non-adherent with adherent

Variable	Lowest-Highest obtainable score	Non-adherence Median (IQR)	Adherence Median (IQR)	Mann-Whitney U test
Stigma Scale				
Distancing Median (IQR)	4.0–16.0	9.0 (6.0–11.5)	8.0 (4.0–11.0)	0.03
Blaming Median (IQR)	4.0–16.0	9.0 (6.0–10.5)	7.0 (5.0–10.0)	0.03
Discrimination Median (IQR)	5.0–20.0	11.0 (8.0–13.5)	10.0 (7.0–13.0)	0.24
Total Stigma	13.0–52.0	27.0 (20.0–35.0)	25.0 (19.0–34.0)	
BMQ Scale				
Necessity Median (IQR)	5.0–25.0	18.0 (16.0–20.0)	18.0 (16.0–20.0)	0.62
Concern Median (IQR)	5.0–25.0	16.0 (14.0–19.0)	16.0 (14.0–18.0)	0.05
Overuse Median (IQR)	4.0–20.0	12.0 (10.0–14.0)	12.0 (10.0–14.0)	0.48
Harm Median (IQR)	4.0–20.0	10.0 (8.0–12.0)	9.0 (8.0–11.0)	0.06
Total BMQ	18.0–90.0	57.0 (52.0–62.0)	55.0 (50.0–60.0)	

*IQR Inter Quartile Range

#### Multivariate logistic regression

Ethnicity and distancing were significantly associated with adherence (Table 4). The sociodemographic factors (age and employment) and the other independent variables (blaming, concern, and harm) were not associated with adherence. Papuans had half the odds of being adherent than non-Papuans (OR = 0.53; 95% CI = 0.32–0.89). Feeling distanced also decreased the odds of being adherent (OR = 0.93; 95% CI = 0.88–0.99).

**Table 4:** Univariate and multivariate analyses on the association of variables with adherence

Characteristics		Univariate OR (95% CI)	*p*-value	Multivariate OR (95% CI)	*p*-value
Age	18–27 years	ref			
	28–37 years	1.67 (0.97–2.87)	0.060		
	≥ 38 years	2.30 (1.26–4.21)	0.006		
Ethnicity	Non-Papuan	ref		ref	
	Papuan	0.55 (0.33–0.92)	0.024	0.53 (0.32–0.89)	0.017
Employment	Unpaid	ref			
	Paid	1.51 (0.96–2.39)	0.077		
Stigma	Distancing	0.94 (0.88–1.00)	0.058	0.93 (0.88–0.99)	0.040
	Blaming	0.93 (0.87–1.00)	0.053		
BMQ	Concern	0.95 (0.88–1.01)	0.155		
	Harm	0.94 (0.87–1.01)	0.135		

*OR Odds Ratio, *CI* Confidence Interval

## Discussion

This cross-sectional study showed that based on self-reporting, more than one-third of PLHIV in Papua, Indonesia were non-adherent. This was lower than in another Indonesian study performed in an urban area [16], but results are difficult to compare because of differences in methods and instruments used. In line with previous studies, the main reasons for non-adherence were forgetfulness, being careless about taking ART, and experiencing adverse effects [25]. Ethnicity and experiencing stigma were significantly associated with non-adherence.

Strength and Limitation.

This was the first study to examine ART adherence in a predominantly heterosexual group of PLHIV living in rural Indonesia where data is scarce. We recruited participants when they came to pick up their medication, in one region in two hospitals in Indonesia with about 10 years of experience to care for HIV patients. Our data may not be generalizable to other settings and regions. For practical reasons, we used hospital health care professionals to recruit patients. We assured patients that participation was voluntary, but some patients may have felt obliged to participate. We used questionnaires and achieved the sample size needed. The study also presents some caveats that need to be discussed. We chose self-reporting to assess adherence. Giving socially desirable answers and recall-bias are among the disadvantages of this method [26]. It was outside the scope of this cross-sectional study to use other methods. The two study hospitals used paper-based medical records and there were insufficient data available to calculate adherence using for example refill rates. Previous studies suggest that in PLHIV, self-reporting is a suitable method [27]. We used the MARS questionnaire that has been developed and validated in schizophrenia patients [18] and the use in PLHIV so far was limited [28]**.** Therefore, a cut-off point for non-adherence has not been defined previously. In our study, PLHIV had to achieve a score of at least 8 out of 10 to be adherent. This is relatively high compared to studies in mental health. We chose this cut-off after using sensitivity analysis. This cut-off point is acceptable to be used since there is small difference between using 80 and 90% adherence due to virological failure [29]. Finally, the available data were insufficient to investigate the association between adherence and clinical proxy outcomes such as CD_4_ counts or viral load. In our setting, those outcomes are infrequently assessed because of lack of resources.

Ethnic group was found to be associated with adherence. Being Papuan increased the likelihood of being non-adherent. One of the reasons for this could be the difficulties in communication between health professionals and PLHIV. Most of the health professionals are non-Papuans and therefore do not speak the local language. This is consistent with another study which revealed that miscommunication between PLHIV and health providers decreased the satisfaction with treatment due to lack of psychological support from health providers [30]. It also caused emotional stress and feeling of discomfort due to loss of self-confidence [31]. The most recognized complaints of PLHIV about physicians are related to communication, not clinical competencies [30]. More work is needed to investigate reasons for non-adherence in Papuans. Our findings highlight the importance to investigate the impact of ethnicity on health care in Indonesia which has 300 ethnic groups, in other regions with a similar mix of ethnicities.

The second result of this study found that stigma, as assessed by the HIV stigma scale, was a significant factor to diminish adherence for PLHIV [8]. Only one of the HIV stigma subscales, feeling more distant, was associated with non-adherence whereas blame and discrimination were not significant. Experiencing stigma could lead to fear to lose one’s companion, and this may result in avoiding treatment hence non-adherence [22]. PLHIV may try to isolate themselves from others in an attempt to protect themselves as a way to cope with the stress of having an HIV infection [32].

In contrast to other studies, the adherent and the non-adherent group were similar in their responses on the BMQ [10]. They perceived a relatively high necessity for ART, but also relatively high concerns and worries about overuse and harmful effects**.** The decision to take ART may not be based on the ratio of risk and benefit of ART alone, but other factors such as stigma were also important. We also found that the difference between the necessity and concern scores were positive for both groups. It means PLHIV were more positive about their medication than they feared to experience adverse effects.

Our results have practical implications for health providers. The first step should be to address communication barriers between health providers and Papuans. Good communication skills may be essential to achieve this. More Papuan social workers from religious institutions may help Papuan PLHIV to solve the miscommunication issues. Overall, more work is needed to disentangle the reasons of why Papuans are more likely to be non-adherent.

Distancing, one type of stigma also needs to be addressed. Clinical strategies may be less successful without changing behavior associated with stigma in society. Awareness about existing stigma may help health professionals to understand patients. Addressing non-adherence may need a tailored intervention based on PLHIV’s needs. Ultimately, health providers are recommended to be active not only to discuss possible adverse effects but also to talk about stigma-related issues with PLHIV. Collaboration between health providers and social workers may encourage the patient’s family to be active in reducing distance by providing more information about HIV. Psychological consultations for PLHIV to learn accepting their HIV status should also be available. Such interventions may lead to increased self-esteem of stigmatized PLHIV to not isolate themselves and actively visit health centers without hesitation.

## Conclusion

In conclusion, self-reported adherence was relatively low in PLHIV in Papua, Indonesia. Ethnicity of being Papuan, and feeling distant from others because of their HIV status were associated with non–adherence. Targeted interventions should be developed to improve adherence in this PLHIV group.

## Additional file

Additional file 1: Appendix BMC Public Health Stigma-Adherence. (DOCX 16 kb)

## Abbreviations

3TC: Lamivudine
AIDS: Acquired Immune Deficiency Syndrome
ART: Antiretroviral Therapy
BMQ: Beliefs about Medicine Questionnaire
CI: Confidence interval
Duv: Duviral
EFV: Efavirenz
FDC: Fixed Dose Combination
FTC: Emtricitabine
HIV: Human Immunodeficiency Virus
IQR: Inter Quartile Range
MARS: Medication Adherence Rating Scale
NEV: Nevirapine
OR: odds ratio
PLHIV: People living with HIV
SD: Standard Deviation
SPSS: Statistical Program for Social Sciences
TDF: Tenofovir
WHO: World Health Organization

## References

[1] UNAIDS (2016). Global AIDS Update.

[2] World Health Organization. Consolidated guidelines on the use of antiretroviral drugs for treating and preventing HIV infection: recommendations for a public health approach. 2013. Available:http://www.who.int/hiv/pub/guidelines/arv2013/. 24716260

[3] World Health Organization (2003). Adherence To Long-Term Therapies.

[4] Becky IBW, Genberg L, Lee Y, Rogers WH. Four types of barriers to adherence of antiretroviral therapy are associated with decreased adherence over time. AIDS Behav. 2015;19(1):85–92. 10.1007/s10461-014-0775-2 PMC4203705 24748240

[5] Goffman Stigma E. Notes on the Management of Spoiled Identity. New York: Simon and Shuster, Inc.; 1963.

[6] Bonnington O, Wamoyi J, Ddaaki W, Bukenya D, Ondenge K, Skovdal M, Renju J, Moshabela M, Wringe A (2017). Changing forms of HIV-related stigma along the HIV care and treatment continuum in sub-Saharan Africa: a temporal analysis. Sex Transm Infect.

[7] Dlamini PS, Wantland D, Makoae LN, Chirwa M, Kohi TW, Greeff M, Naidoo J, Mullan J, Uys LR, Holzemer WL (2009). HIV stigma and missed medications in HIV-positive people in five African countries. AIDS Patient Care STDs.

[8] Rueda S, Mitra S, Chen S, Gogolishvili D, Globerman J, Chambers L, Wilson M, Logie CH, Shi Q, Morassaei S, Rourke SB (2016). Examining the associations between HIV-related stigma and health outcomes in people living with HIV/AIDS: a series of meta-analyses. BMJ Open.

[9] Horne R, Weinman J, Hankins M (1999). The beliefs about medicines questionnaire: the development and evaluation of a new method for assessing the cognitive representation of medication. Psychol Health.

[10] Horne R, Chapman SCE, Parham R, Freemantle N, Forbes A, Cooper V. Understanding patients’ adherence-related beliefs about medicines prescribed for long-term conditions: a meta-analytic review of the necessity-concerns framework. PLoS One. 2013;8(12):e80633. 10.1371/journal.pone.0080633 PMC3846635 24312488

[11] Wasti SP, Van Teijlingen E, Simkhada P, Randall J, Baxter S, Kirkpatrick P, Gc VS (2012). Factors influencing adherence to antiretroviral treatment in Asian developing countries: a systematic review. Trop Med Int Heal.

[12] Direktorat Jenderal Pencegahan dan Pengendalian Penyakit (2017). Laporan Perkembangan HIV/AIDS 7 Penyakit Menular Seksual (PIMS) Triwulan I Tahun 2017.

[13] Dinas Kesehatan Provinsi Papua (2017). Profil Kesehatan Provinsi Papua Tahun 2016.

[14] Indonesia National AIDS Commission Indonesia. Global AIDS response Progress reporting Indonesia country Progress report 2014. 2014;1:35–110.

[15] Li Y, Hershow R, Irwanto, Praptoraharjo I, Setiawan M, Levy J (2014). Factors associated with symptoms of depression among injection drug users receiving antiretroviral treatment in Indonesia. J AIDS Clin Res.

[16] Weaver ERN, Pane M, Wandra T, Windiyaningsih C, Herlina, Samaan G (2014). Factors that influence adherence to antiretroviral treatment in an urban population, Jakarta, Indonesia. PLoS One.

[17] E. Sianturi, K. Taxis, D. Perwitasari, and A. Islam. Association between Stigma, Beliefs about Medicines and Adherence to Antiretroviral Therapy: A Cross Sectional Study in People Living with HIV (PLHIV). In: European Drug Utilisation Research Group Conference Glasgow 2017, 2017, no. November, p. 54.

[18] Thompson K, Kulkarni J, Sergejew AA (2000). Reliability and validity of a new medication adherence rating scale (MARS) for the psychoses. Schizophr Res.

[19] Sowell RL, Lowenstein A, Moneyham L, Demi A, Mizuno Y, Seals BF (1997). Resources, stigma, and patterns of disclosure in rural women with HIV infection. Public Health Nurs.

[20] Fialko L, Garety PA, Kuipers E, Dunn G, Bebbington PE, Fowler D, Freeman D (2008). A large-scale validation study of the medication adherence rating scale (MARS). Schizophr Res.

[21] Farber EW, Shahane AA, Brown JL, Campos PE (2014). Perceived stigma reductions following participation in mental health services integrated within community-based HIV primary care. AIDS Care - Psychol Socio-Medical Asp AIDS/HIV.

[22] Emlet CA (2005). Measuring stigma in older and younger adults with HIV/AIDS: an analysis of an HIV stigma scale and initial exploration of subscales. Res Soc Work Pract.

[23] Epstein J, Santo RM, Guillemin F. A review of guidelines for cross-cultural adaptation of questionnaires could not bring out a consensus. J Clin Epidemiol. 2015;68:435–41. 10.1016/j.jclinepi.2014.11.021 25698408

[24] Bursac Z, Gauss CH, Williams DK, Hosmer DW (2008). Purposeful selection of variables in logistic regression. Source Code Biol Med.

[25] Shubber Z, Mills EJ, Nachega JB, Vreeman R, Freitas M, Bock P, Nsanzimana S, Penazzato M, Appolo T, Doherty M, Ford N (2016). Patient-reported barriers to adherence to antiretroviral therapy: a systematic review and meta-analysis. PLoS Med.

[26] Elsievers M, Wettermark B, Almasdóttir AB, Andersen M, Benko R, Bennie M, Eriksson I, Godman B, Krska J, Poluzi E, Taxis K, Vlahović-Palćevksi V, Stichele V (2016). Drug Utiliation Research: Methods and Application.

[27] Simoni JM, Pearson CR, Pantalone DW, Marks G, Crepaz N (2006). Efficacy of interventions in improving highly active antiretroviral therapy adherence and HIV-1 RNA viral load: a meta-analytic review of randomized controlled trials. J Acquir Immune Defic Syndr.

[28] Nguyen TMU, La Caze A, Cottrell N (2014). What are validated self-report adherence scales really measuring?: a systematic review. Br J Clin Pharmacol.

[29] Rosenblum M, Deeks SG, van der Laan M, Bangsberg DR (2009). The risk of virologic failure decreases with duration of HIV suppression, at greater than 50% adherence to antiretroviral therapy. PLoS One.

[30] Ha JF, Longnecker N, Hons M, Anat D, Longnecker N, Jennifer Fong Ha P, Anat DS, Longnecker N (2010). Doctor-Patient Communication: A Review. Ochsner J.

[31] Scheppers E, van Dongen E, Dekker J, Geertzen J, Dekker J (2006). Potential barriers to the use of health services among ethnic minorities: a review. Fam Pract.

[32] Zhang YX, Golin CE, Jin B, Emrick CB, Nan Z, Li MQ (2014). Coping strategies for HIV-related stigma in Liuzhou, China. AIDS Behav.

