# Supplementary material for: The association between ethnicity, stigma, beliefs about medicines and adherence in people living with HIV in a rural area in Indonesia
Source: BMC Public Health. 2019 Jan 11;19:55. doi: 10.1186/s12889-019-6392-2 (PMC6330480; doi:10.1186/s12889-019-6392-2)
Supplement: Supplementary file 1 — Appendix BMC Public Health Stigma-Adherence. (DOCX 16 kb) [file 12889_2019_6392_MOESM1_ESM.docx]

The sensitivity and specificity analysis of cut off for adherence

Likelihood

| Cut of 70% | Non-adherence | Adherence | Total |
| --- | --- | --- | --- |
| Female | 39 | 165 | 222 |
| Male | 19 | 108 | 109 |
| Total | 58 | 273 | 331 |

Sensitivity (Papua): 39/58 =0.67

Specificity (Non-Papua): 108/273=0.39

**Likelihood:** 0.67/(1-0.39) = 1.099

| Cut of 80% | Non-adherence | Adherence | Total |
| --- | --- | --- | --- |
| Female | 75 | 129 | 222 |
| Male | 38 | 89 | 109 |
| Total | 113 | 218 | 331 |

Sensitivity (Papua): 75/113 = 0.66

Specificity (Non-Papua): 89/218 = 0.41

**Likelihood:** 0.66 /(1-0.41) **= 1.119**

| Cut of 90% | Non-adherence | Adherence | Total |
| --- | --- | --- | --- |
| Female | 119 | 85 | 222 |
| Male | 70 | 57 | 109 |
| Total | 189 | 142 | 331 |

Sensitivity (Papua): 119/189 = 0.63

Specificity (Non-Papua): 57/142 = 0.40

Likelihood: 0.67 /(1-0.40) = 1.117
